# Adoption and Integration of “Complete Anatomy” in Early‐Year Medical Education: Student and Staff Perspectives

**DOI:** 10.1002/ca.70060

**Published:** 2025-12-10

**Authors:** Noor Al‐Antary, Islam Muflahi, John M. Delieu, Sami A. Al‐Ani, Claire J. Stocker

**Affiliations:** ^1^ Aston Medical School Aston University Birmingham UK

**Keywords:** 3D anatomy, complete anatomy, curriculum integration, digital learning, medical education, student engagement

## Abstract

Digital anatomy platforms are used in undergraduate healthcare education, but their integration into early curricula varies and often lacks alignment with instructional design. This study evaluates the implementation of Complete Anatomy, a three‐dimensional anatomy platform, within an early‐year medical curriculum. A mixed‐methods design was used to collect student and staff data through surveys and focus groups. Student responses showed selective use focused on visualization tools, with limited engagement with quizzes and annotation features. Staff reported low familiarity and minimal use in teaching. Reported barriers included technical instability, navigation difficulty, and lack of integration with learning outcomes. Students and staff proposed curriculum actions to support platform use, including onboarding during induction, guided tasks in tutorials, tutor modeling, and alignment with block content. These actions respond to operational constraints and support structured adoption. The findings provide a framework for platform‐specific implementation that may improve consistency of use and reduce cognitive burden. This approach supports integration of digital anatomy tools into early medical education and may inform institutional strategies for resource adoption across disciplines.

## 1 Introduction

Anatomy education has traditionally relied on cadaveric dissection, printed atlases, and physical models. These approaches remain widely used and continue to support the development of spatial understanding and anatomical knowledge (Preece et al. 2013). The decline in the availability of cadavers, the cost and ethical issues surrounding their use, combined with the increasing availability of digital platforms, has prompted institutions in recent years to explore alternative methods, including three‐dimensional (3D) anatomy applications, virtual reality, and simulation‐based tools. These developments reflect broader trends in technology‐enhanced learning, which aim to improve accessibility, engagement, and visualization in anatomy education (Wang et al. 2024).

Complete Anatomy is a commercially available 3D anatomy platform developed by 3D4Medical (Elsevier). It enables users to manipulate anatomical models, isolate structures, and access embedded videos and quizzes. The platform is available on desktop and mobile devices and has been adopted in several undergraduate medical programs (3D4Medical 2025). Its use forms part of a wider shift toward the increasing use of digital resources in early medical training, particularly where institutions seek to supplement or replace traditional methods due to a variety of reasons, including cost, access, and logistics.

Evidence regarding the educational benefits of 3D anatomy platforms remains mixed. Meta‐analyses and controlled studies have reported improvements in the acquisition of factual anatomy knowledge, spatial understanding, and learner satisfaction (Yammine and Violato 2015), though effects on examination scores and the time required to answer questions are inconsistent (Wang et al. 2024). A comparative study found that MRI assessment scores were significantly higher in students utilizing a physical model compared with students using textbooks or a 3D computer model, with no significant difference between the textbook and 3D computer model groups. This suggested that physical models may support better identification of structures on radiological images than digital models, with implications for visuospatial learning of complex anatomical architecture (Preece et al. 2013). These findings indicate that digital platforms may have a role in complementing but not necessarily replacing established teaching methods.

Studies looking at the effectiveness of anatomy education sometimes mix across levels of anatomical complexity, learner stage (undergraduate versus postgraduate), and educational context (human vs. veterinary), which can lead to inaccurate or overly broad comparisons. The present study focuses on the platform's contribution and contextual relevance within early‐years undergraduate human anatomy education.

Not all digital anatomy tools are the same. A limitation of prior research is the tendency to group all three‐dimensional virtual anatomy (3DVA) platforms under a single category, despite substantial differences in software features, fidelity, and interactivity. This aggregation risks obscuring platform‐specific effectiveness and misrepresenting potential educational benefits.

Student uptake and adoption of different tools depend on multiple factors. Surveys have shown that the uptake of digital tools is shaped by several factors, including perceived usefulness, ease of use, enjoyment, and stimulation (Albrecht et al. 2013; Singer et al. 2024). The Technology Acceptance Model has been applied to anatomy education to explain adoption patterns, highlighting the influence of enjoyment, self‐efficacy (an individual's opinion as to whether they can use a piece of equipment), and subjective/social norms (an individual's perception of the extent to which they think other students think they should use a piece of equipment) (Singer et al. 2024). These findings suggest that platform design and curriculum integration play a critical role in determining sustained use.

Despite the widespread adoption of the Complete Anatomy platform in medical education, few studies have systematically examined its implementation within early‐year curricula (Smith et al. 2022). Existing literature has primarily focused on student performance outcomes or overall satisfaction, with limited exploration of usability, navigation, and alignment with pedagogical practices. There remains a lack of triangulated evaluations incorporating both student and staff perspectives and translating identified barriers into curriculum‐level recommendations. This evaluation focuses on the implementation of a single platform, Complete Anatomy, within a defined early‐year medical curriculum. It triangulates student and staff data to identify operational barriers and translate them into curriculum‐level actions. The aim is not to assess general satisfaction or performance outcomes, but to inform platform‐specific integration strategies that respond to usability constraints, pedagogical gaps, and institutional needs. The findings support a framework for structured adoption of digital anatomy tools in early medical education.

## 2 Methods

### 2.1 Study Design

A mixed‐methods design was used to evaluate the implementation and perceived impact of the Complete Anatomy platform in early‐year anatomy education. Data were collected through student and staff surveys and student focus groups. The aim was to triangulate quantitative and qualitative findings to identify usage patterns, barriers to engagement, and curriculum integration needs.

### 2.2 Participants

Participants included first‐ and second‐year students enrolled in a core anatomy course across three programs: medicine, nursing, and physician associate studies. A total of 100 students were invited to complete the survey, with 45 responses received (response rate: 45%). Six students volunteered to participate in focus group discussions. Nine teaching staff involved in direct anatomy teaching at the Aston Medical School were invited to complete the staff survey, with full participation (response rate: 100%).

Students are taught human anatomy in their early years using a blended approach of lectures and modified Team Based Learning tutorials with some exposure to cadaveric material. Students are provided with an iPad with the Complete Anatomy platform pre‐loaded at the start of their course.

### 2.3 Intervention

Students were provided with access to the Complete Anatomy platform as part of their anatomy education. The platform is utilized during group work tutorials and peer‐led presentations. Students were also encouraged to use the platform independently to explore 3D anatomical models and engage in self‐directed study.

### 2.4 Data Collection

Two semi‐structured focus groups were conducted with six students. Discussions explored perceived benefits, limitations, and usability of the platform. Sessions were audio‐recorded and transcribed verbatim. The student survey included Likert‐scale items assessing ease of use, perceived learning benefit, engagement, navigation, and integration with existing teaching methods. Open‐ended questions captured challenges and suggestions for improvement. The staff survey included parallel Likert‐scale items focused on pedagogical value, feature familiarity, and curriculum integration, alongside open‐text responses on barriers and enhancement strategies.

### 2.5 Data Analysis

Quantitative survey data were analyzed using descriptive statistics to summarize usage frequency, satisfaction levels, and reported challenges. Qualitative data from focus group transcripts and open‐ended survey responses were analyzed thematically, following the six‐phase framework described by Braun and Clarke (2006), to identify recurring patterns and interpretive insights.

## 3 Results

### 3.1 Student Survey Data

#### 3.1.1 Overview

Forty‐six students completed the survey. Respondents were predominantly aged 18–24 and held A‐level or equivalent qualifications. Tables 1a and 1b present the full distribution of responses and categorical summaries. The narrative below synthesizes key patterns and implications.

**TABLE 1a:** Quantitative survey responses.

Item	Response options	Percentage
Age	18–24	96%
	25–34	4%
Usage frequency	Daily	9%
	Weekly	48%
	Monthly	20%
	Rarely	22%
	Never	2%
Engagement level	Very engaging	39%
	Moderately engaging	41%
	Slightly engaging	20%
	Not engaging at all	0%
Ease of navigation	Very difficult	11%
	Difficult	28%
	Neither easy nor difficult	33%
	Easy	24%
	Very easy	4%
Leads to improved understanding of anatomy	Yes	72%
	No	9%
	Not sure	20%
Leads to increase study time compared to traditional methods	Strongly agree	35%
	Disagree	33%
	Strongly disagree	17%
	Not applicable	9%
Would recommend to peers	Strongly agree	30%
	Agree	52%
	Disagree	11%
	Strongly disagree	0%
	Not applicable	7%
Most helpful aspect	3D visualizations	78%
	Detailed anatomical structures	15%
	Interactive quizzes	4%
	Other	2%

**TABLE 1b:** Barriers, preferences, and support needs.

Item	Response options	Percentage or summary
Reasons for not using complete anatomy	Preference for other study methods	28%
	Difficulty understanding how to use the platform	30%
	Lack of access	2%
	Not aware of its existence	2%
	Other	28%
Challenges faced while using the platform	Yes	54%
	No	26%
	Not sure	20%
Reported challenges	Free‐text responses	Technical issues, navigation difficulties, content relevance, lack of guidance, time spent
Interest in using Complete Anatomy if more training and support are provided	Strongly agree	41%
	Agree	48%
	Disagree	7%
	Strongly disagree	0%
	Not applicable	4%
Other alternative study methods	Free‐text responses	Textbooks, videos, Teach Me Anatomy, Kenhub, YouTube, lecture slides, anatomy models, flashcards, 3D printed models
Perceived benefits of technology‐enhanced learning	Free‐text responses	High‐quality visualizations, portability, accessibility, interactive learning, enhanced engagement

#### 3.1.2 Usage Frequency and Engagement

Most students reported weekly or monthly use of the Complete Anatomy platform, with a minority using it daily or not at all. Engagement ratings were generally positive, though a substantial proportion described only moderate or slight engagement. These patterns suggest routine but not embedded use. See Table 1a for full usage and engagement distributions.

#### 3.1.3 Barriers and Usability

Students who did not use the platform cited difficulty understanding its functions and preference for other resources. Navigation was frequently described as difficult, and awareness of embedded features such as quizzes and videos was low. Free‐text responses repeatedly identified technical issues, lack of guidance, and time inefficiency. These findings align with focus group accounts and suggest that usability remains a limiting factor. See Table 1b for barrier categories and reported challenges.

#### 3.1.4 Learning Impact and Study Behavior

Most students reported improved understanding of anatomy when using the platform. However, responses were mixed regarding time spent and study efficiency. A third of students disagreed that the platform increased the time they spent studying anatomy compared to traditional methods, and nearly one fifth strongly disagreed. These findings indicate that despite a perceived benefit in using the platform, this does not necessarily translate into prolonged and sustained use. An explanation maybe that students perceive that their learning is more efficient. See Table 1a for impact and study time responses.

#### 3.1.5 Support Needs and Preferences

Interest in structured support was high, with nearly all students expressing willingness to engage more with the platform if more guidance was provided. Students described a wide range of alternative study methods, including textbooks, videos, anatomy models, and online platforms. These preferences reflect a diverse resource landscape and suggest that the Complete Anatomy platform is used selectively rather than universally. See Table 1b for support interest and alternative methods.

#### 3.1.6 Perceived Value and Recommendation

Students identified 3D visualizations as the most helpful feature, with limited endorsement of quizzes or structural detail. Most students would recommend the platform to peers, though endorsement was stronger among regular users. These findings suggest that perceived value is concentrated in visual features, with other functions underused or undervalued. See Table 1a for feature ratings and recommendation responses.

### 3.2 Focus Groups Data

**FIGURE 1:**
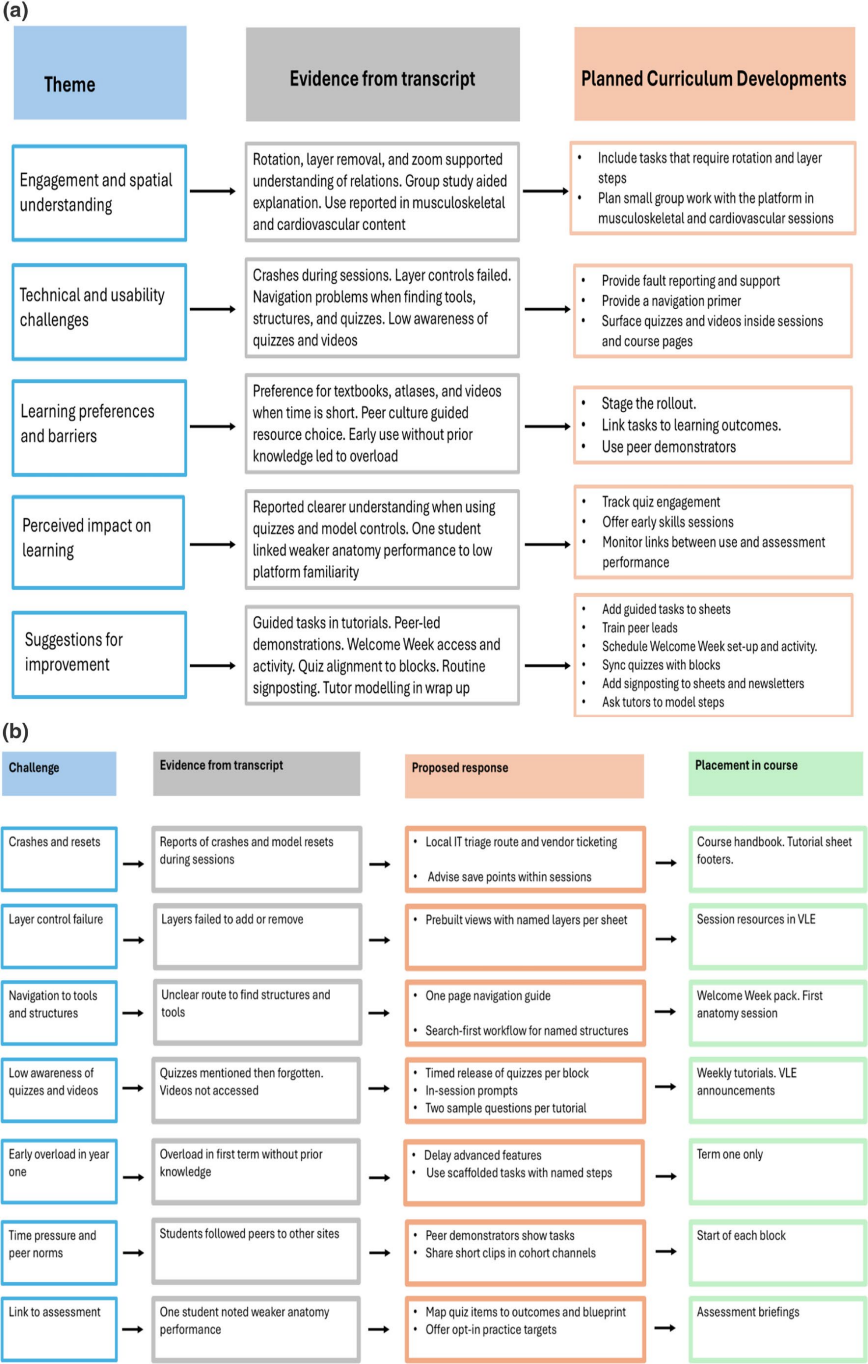
(a) Themes, evidence, and implications. (b) Student proposals for integration and corresponding action.

Semi‐structured interviews with six students produced five themes. These were engagement and spatial understanding, technical and usability challenges, learning preferences and barriers, perceived impact on learning, and suggestions for improvement. Figure 1a summarizes all themes with evidence and implications.

#### 3.2.1 Engagement and Spatial Understanding

In focus groups, students reported that their engagement in anatomy sessions increased when they used the Complete Anatomy platform. They described using it during musculoskeletal and cardiovascular teaching sessions. Platform features such as rotation, layer control, and zoom supported students' understanding of spatial relationships between anatomical structures. Additionally, using the platform during group study facilitated peer explanations and discussion. See Figure 1a.

#### 3.2.2 Technical and Usability Challenges

Some students reported application crashes, layer control problems, and difficulty locating tools, quizzes, and named structures. Awareness of embedded quizzes and videos was low. These challenges, particularly difficulties finding content, interrupted study for some students and occasionally led them to discontinue platform use. See Figure 1a.

#### 3.2.3 Learning Preferences and Barriers

When time was limited, students described a preference for textbooks, atlases, and videos or when peers recommended these resources. Students tended to use the Complete Anatomy platform when tasks explicitly required it or when peers demonstrated how to use it. Students reported feeling overloaded when using the platform early in their first year of studies without prior anatomical knowledge. Students framed the platform as a supplement rather than a primary method of studying anatomy. See Figure 1a.

#### 3.2.4 Perceived Impact on Learning

Students who engaged with quizzes and model manipulation reported a clearer understanding of structures and relations in musculoskeletal and cardiovascular content. One student reported weaker anatomy performance and suggested that better familiarity with the platform might have improved outcomes. Students linked impact to prior knowledge, stable access, and structured guidance within teaching. See Figure 1a.

#### 3.2.5 Student Proposals for Enhanced Integration

Students proposed guided tasks within tutorials that require use of the platform to locate structures and information. They also proposed peer‐led demonstrations and short instructional videos. Students proposed that they should be granted access and be set a practical activity using the platform in Welcome Week, prior to the start of formal teaching. Students proposed alignment of quiz content with current blocks and more signposting in tutorials and newsletters to quizzes and tools on the platform. They also suggested that tutors demonstrate the use of the platform during the wrap‐up at the end of tutorials. See Figure 1b.

### 3.3 Staff Survey Data

#### 3.3.1 Overview

Nine staff members completed the survey. Respondents were predominantly aged 35–54 and held university qualifications in medicine and science, including MBBS, MD, PhD, and MSc. Tables 2a and 2b present full response distributions and categorical summaries.

**TABLE 2a:** Quantitative staff survey responses.

Item	Response options	Percentage
Age	25–34	11%
	35–45	56%
	45–54	22%
	55 or older	11%
Usage frequency	Daily	0%
	Weekly	33%
	Monthly	22%
	Rarely	22%
	Never	22%
Familiarity with features	Very familiar	0%
	Familiar	44%
	Slightly familiar	44%
	Not familiar at all	11%
Ease of navigation	Very difficult	11%
	Difficult	11%
	Neither difficult nor easy	76%
	Easy	11%
	Very easy	0%
Interface user‐friendliness	Strongly agree	0%
	Agree	67%
	Disagree	22%
	Strongly disagree	0%
	Not applicable	11%
Perceived improvement in students' understanding	Yes	44%
	Not sure	56%
Recommend to peers	Strongly agree	22%
	Agree	67%
	Disagree	0%
	Strongly disagree	0%
	Not applicable	11%
Most helpful aspect	3D visualizations	78%
	Detailed anatomical structures	11%
	Interactive quizzes	0%
	Comparative anatomy tools	0%
	Other	11%

**TABLE 2b:** Barriers, support needs, and enhancement priorities.

Item	Response options or summary	Percentage or category
Reasons for not using complete anatomy	Lack of access	22%
	Preference for other teaching methods	22%
	Difficulty understanding how to use it	0%
Interest in using with structured support	Strongly agree	44%
	Agree	44%
	Disagree	11%
	Strongly disagree	0%
	Not applicable	0%
Suggested enhancements (free text)	Upload own content	Categorical
	Clinically relevant scenarios	Categorical
	More practice questions	Categorical
	Improved account access and verification	Categorical
	Enhanced search and quiz creation	Categorical
	More Clinical imaging modalities	Categorical
	Augmented Reality feature	Categorical
	Compartment‐specific resources	Categorical
Challenges with technology‐enhanced learning	Integration with current courses	Categorical
	Training and familiarity	Categorical
	Navigation and accessibility	Categorical
	Digital literacy variation	Categorical
Perceived benefits of technology‐enhanced learning	Visual understanding	Categorical
	Accessibility and portability	Categorical
	Interactive and engaging methods	Categorical
	Innovation in teaching approaches	Categorical

#### 3.3.2 Usage and Familiarity

One third of staff reported weekly use of the Complete Anatomy platform. The remainder used it monthly, rarely, or not at all. Familiarity with platform features was limited: none reported being very familiar, and nearly half described only slight familiarity. These findings suggest a possible lack of training but also that use is sporadic and not embedded in routine teaching practice. See Table 2a.

#### 3.3.3 Navigation and Interface

Navigation was described as difficult or neutral by most respondents, and none rated it as very easy. Two‐thirds agreed that the interface was user‐friendly, though a minority disagreed. These findings suggest that while the interface is broadly acceptable, it does not actively support confident use. See Table 2a.

#### 3.3.4 Barriers to Use

Staff who did not use the platform cited a lack of access and a preference for other teaching methods. No respondents reported difficulty understanding how to use it. Free‐text responses identified technical limitations, interface complexity, and a lack of integration with existing workflows. See Table 2b.

#### 3.3.5 Perceived Student Impact

Four staff members (44%) reported perceived improvement in students' anatomical understanding by use of the platform by staff, students, or both. The remainder were unsure. No respondents reported a negative impact. These findings suggest cautious optimism but limited direct observation. See Table 2a.

#### 3.3.6 Support Needs and Enhancement Priorities

Most staff expressed interest in using the platform with structured support. Suggested enhancements to the platform included having the ability to upload content, improved search and quiz functions, compartment‐specific resources, and integration of clinical scenarios. These priorities reflect a desire for greater control, relevance, and alignment with teaching aims. See Table 2b.

#### 3.3.7 Technology‐Enhanced Learning

Staff described the benefits of technology‐enhanced learning as enhanced visual understanding, accessibility, and engagement. Reported challenges included integration with current courses, training and familiarity, navigation issues, and digital literacy variation in staff. These perceptions align with student feedback and suggest that platform adoption depends on structured support and curriculum alignment. See Table 2b.

#### 3.3.8 Recommendation to Peers

Most staff would recommend the platform to other educators, though none strongly endorsed it. Perceived value was concentrated in 3D visualizations, with limited endorsement of other features. See Table 2a.

## 4 Discussion

Triangulated data from students and staff indicate that the Complete Anatomy platform is used selectively, with engagement concentrated around model manipulation tools. Students reported perceived improvements in spatial understanding, particularly when using rotation, zoom, and layer controls. However, technical instability, navigation difficulties, and low awareness of embedded features constrained broader use. Staff responses reflected low familiarity with the platform and minimal integration into teaching, despite recognizing its potential value. These findings align with prior evidence that 3D anatomy tools can support spatial reasoning and learner satisfaction, though effects on examination performance and study efficiency remain inconsistent (Yammine and Violato 2015; Wang et al. 2024). The platform's interactive models facilitated exploratory learning, consistent with constructivist learning theory, which emphasizes active engagement with materials as a basis for knowledge construction (Vygotsky 1978). From a cognitive load perspective, multimodal presentation may reduce extraneous load and support germane processing (Paas et al. 2003), though usability constraints may increase cognitive burden for some users. Assumptions about student digital fluency may not hold uniformly. Singer et al. (2024) reported that students navigating unfamiliar anatomical content while learning new technology may experience greater cognitive load than staff, who engage with the platform from a position of subject expertise. This distinction highlights the need for staged onboarding and scaffolded tasks that account for both technological and anatomical learning curves. It also suggests that platform adoption cannot rely solely on availability or presumed digital competence.

Staff responses identified gaps in digital literacy and curriculum alignment. These findings reflect broader concerns about faculty readiness and the need for structured frameworks to support the use of simulation and digital tools in anatomy education (Kneebone 2003). Without integration into learning outcomes and assessment, platforms risk fragmented implementation and underuse. However, the staff sample was limited to a single institution and may not reflect broader patterns of adoption or training provision. Similarly, student survey responses were self‐reported and may not correspond to actual time on task or depth of engagement.

**FIGURE 2:**
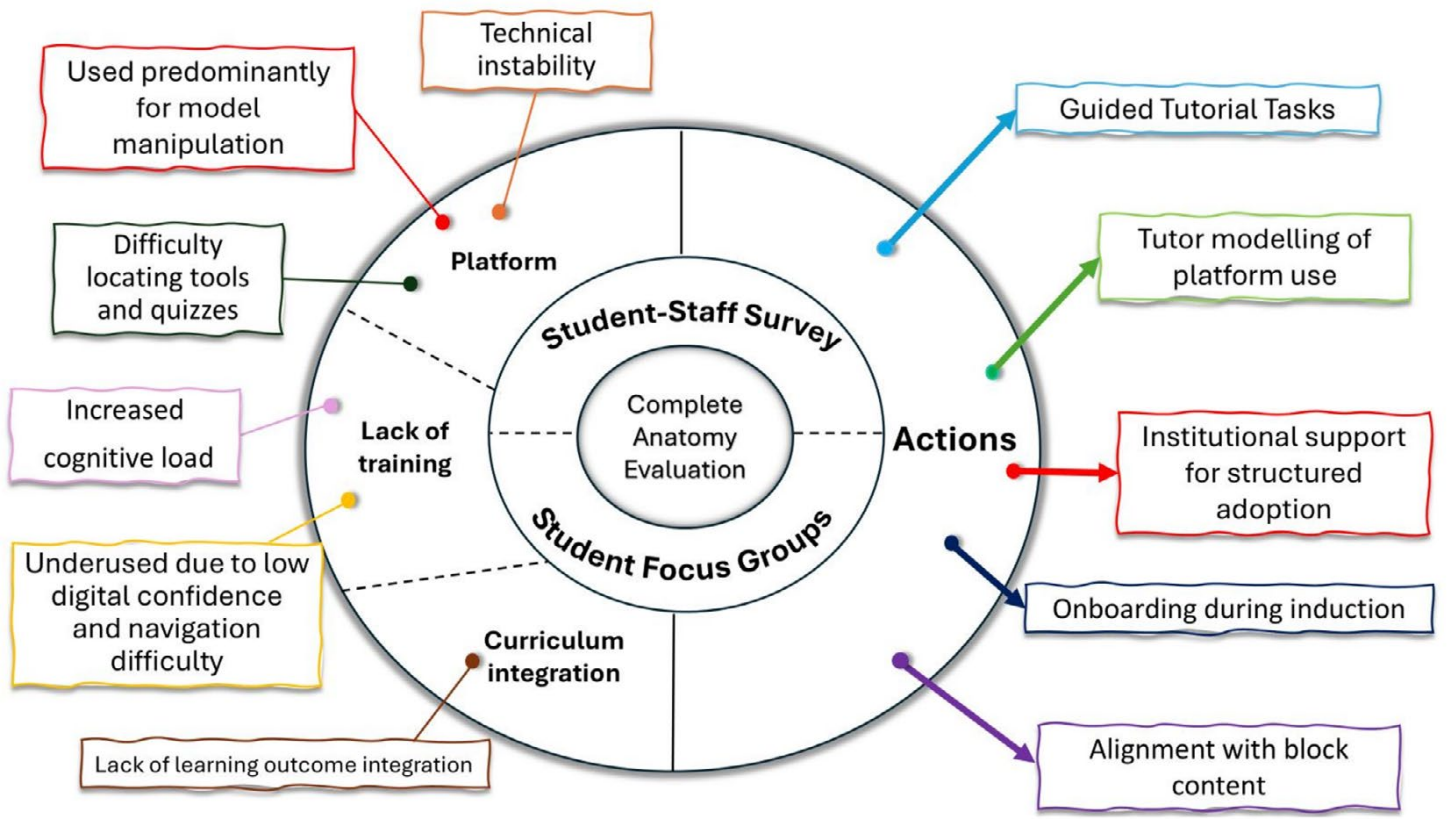
Central node represents the evaluation focus: Implementation of the Complete Anatomy platform in early‐year anatomy education. Encircling node represents methods of evaluation: Student‐staff survey and student focus groups. Left half of the outer node summarizes triangulated findings from student and staff data, including selective use for model manipulation, technical instability, difficulty locating tools and quizzes, lack of training, and lack of learning outcome integration. Right half of the outer node represents curriculum actions proposed by participants: Guided tutorial tasks, tutor modeling, institutional support, onboarding during induction, and alignment with block content.

Students and staff proposed curriculum‐level actions including induction‐based onboarding, guided tutorial tasks, tutor modeling, and institutional support for structured adoption. These actions are summarized in Figure 2 and reflect a direct mapping from reported barriers to curriculum interventions. The proposed actions are consistent with instructional design principles that link activities to assessment and support sustained engagement (Biggs 1996). While grounded in participant feedback, their effectiveness has not yet been evaluated and may require adaptation to different institutional contexts.

The inclusion of students from medicine, nursing, and physician associate programs supports broader applicability across disciplines. Shared use of anatomy platforms may also facilitate interprofessional learning, consistent with guidance from the Centre for the Advancement of Interprofessional Education (Barr et al. 2016). However, the study did not assess interprofessional outcomes directly, and further work is needed to determine whether shared platforms contribute meaningfully to collaborative learning.

Implementation strategies that address platform‐specific constraints, stakeholder familiarity, and curriculum alignment may improve consistency of use and reduce cognitive burden. Figure 2 presents a visual summary of triangulated findings and proposed curriculum actions, supporting a replicable framework for embedding non‐immersive 3D platforms into early medical education and promoting coherent adoption across disciplines.

## 5 Conclusion

Triangulated evaluation of the Complete Anatomy platform across students and staff identified selective engagement, usability constraints, and limited pedagogical integration. While visualization tools supported spatial understanding, broader use was constrained by technical issues, navigation difficulties, and low awareness of platform features. Staff responses indicated gaps in digital literacy and curriculum alignment, with limited use in teaching despite recognition of potential value. Mapped curriculum actions, including induction‐based onboarding, guided tutorial tasks, tutor modeling, and alignment with learning outcomes, offer a structured approach to embedding digital anatomy platforms in early medical education. These actions respond directly to reported barriers and support more consistent use across disciplines. The findings provide a framework for platform‐specific implementation that can inform institutional strategies for digital resource adoption in anatomy education.

## Funding

This work was supported by Aston University Teaching and Research.

## Ethics Statement

The study was approved by Aston University Ethics Committee, Ref: HLS21151.

## Data Availability

The data that support the findings of this study are openly available.
